# An Acute Peripheral‐Appearing Vestibular Syndrome Followed by Probable Vestibular Migraine: A Case Report

**DOI:** 10.1002/ccr3.73846

**Published:** 2026-10-30

**Authors:** Xiaocui Kang, Shiyu Jiang, Siyang Dai, Ying Wu, Zhenyu Wei, Ping Zhong

**Affiliations:** ^1^ Department of Neurology Shidong Hospital Shanghai China; ^2^ University of Shanghai for Science and Technology Shanghai China

**Keywords:** acute unilateral vestibulopathy, acute vestibular syndrome, probable vestibular migraine, vestibular neuritis, video head impulse test, videonystagmography

## Abstract

Peripheral‐appearing findings during an acute vestibular syndrome do not necessarily establish the etiology of subsequent recurrent vestibular episodes. The emergence of recurrent vertigo with migraine‐associated features should prompt diagnostic reassessment. Serial vestibular testing may document evolving vestibular function but should be interpreted in conjunction with the longitudinal clinical phenotype.

## 1 Introduction

Vertigo and dizziness are common neurological complaints. Among patients presenting with acute vestibular syndrome (AVS), distinguishing peripheral vestibular disorders from central causes, particularly posterior circulation stroke, can be challenging, especially when vertigo occurs without obvious focal neurological deficits [1, 2, 3]. Bedside oculomotor examination, vestibular function testing, and neuroimaging provide complementary information in the evaluation of these patients. However, the diagnostic formulation may require reassessment when the temporal pattern or associated clinical features evolve during follow‐up [2, 3, 4].

Vestibular migraine (VM) is a heterogeneous disorder in which both central and peripheral vestibular abnormalities have been reported [5]. Vestibular laboratory findings may vary between patients and across different phases of the disorder, and abnormalities of nystagmus, vestibulo‐ocular reflex function, and other oculomotor measures may occur during symptomatic episodes [5, 6, 7]. However, these findings are not specific to VM, and vestibular testing is primarily useful as part of the overall clinical assessment and differential diagnosis rather than as a standalone diagnostic marker.

Acute unilateral vestibulopathy (AUVP), commonly termed vestibular neuritis (VN) when no acute audiological symptoms are present, typically presents as an acute, sustained vestibular syndrome associated with unilateral vestibular hypofunction [8, 9, 10, 11]. In contrast, VM and probable vestibular migraine (pVM) are characterized by recurrent vestibular episodes defined primarily by their clinical pattern and associated migraine history or migraine features. Importantly, overlap in vestibular and oculomotor findings may occur, and changes in serial vestibular testing should therefore be interpreted in the context of the evolving clinical phenotype rather than used in isolation to distinguish these disorders [6, 7, 11].

We report a 64‐year‐old woman who initially presented with an acute peripheral‐appearing vestibular syndrome compatible with right AUVP/VN and subsequently developed recurrent vestibular episodes associated with photophobia and phonophobia, considered consistent with the clinical framework for pVM. Serial vestibular assessment demonstrated changes in spontaneous nystagmus and vHIT findings over time. This case illustrates the diagnostic complexity of evolving vestibular syndromes and highlights the importance of longitudinal clinical reassessment when the temporal pattern and associated features change.

## 2 Case Presentation and Examination

### 2.1 Patient Information and Initial Presentation

A 64‐year‐old woman was admitted on August 16, 2025, because of acute‐onset vertigo accompanied by nausea and vomiting for 1 day. At approximately 12:00 on August 15, following travel, she developed sudden‐onset vertigo described as a marked swaying sensation. The vertigo persisted for several hours and was accompanied by nausea and multiple episodes of vomiting. She remained fully conscious and reported no headache or visual aura. The vertigo was not clearly triggered by positional changes. She denied hearing loss, tinnitus, aural fullness, or limb weakness. Decades earlier, she had experienced a single brief episode of rotational vertigo lasting approximately 10 min that resolved with bed rest.

Her medical history included hypertension, for which she had previously received antihypertensive treatment but was not taking medication at the time of admission, and Type 2 diabetes mellitus for 15 years, complicated by diabetic retinopathy diagnosed 1 year earlier. She had no notable history of infectious disease, surgery, substance use, motion sickness, or family history of vertigo.

### 2.2 Examination on Admission

On August 16, her heart rate was 65 beats/min and blood pressure was 130/80 mmHg. She was alert and had clear speech. The pupils were equal (3 mm) and reactive to light. Bedside examination demonstrated horizontal spontaneous nystagmus with a leftward fast phase, which was more prominent on left gaze; no clear nystagmus was observed on right gaze. The bedside head‐impulse test could not be reliably interpreted because of suboptimal cooperation. The remaining cranial nerve examination was unremarkable. Limb strength was Grade V bilaterally, superficial and deep sensation were symmetric, and finger‐to‐nose and heel‐to‐shin testing showed no dysmetria. The Romberg test was negative, and bilateral Babinski signs were absent. The neck was supple, with no meningeal signs.

Routine blood, urine, and stool examinations; liver and renal function tests; serum electrolytes; cardiac enzymes; tumor markers; thyroid function tests; electrocardiography; Holter monitoring; and echocardiography revealed no clinically significant abnormalities.

### 2.3 Initial Neuroimaging and Vestibular Assessment

Emergency head CT performed on August 16 showed no definite intracranial hemorrhage or space‐occupying lesion. A focal area of mildly decreased attenuation was noted in the right cerebellar hemisphere, and follow‐up imaging was recommended (Figure 1A). Brain MRI performed on August 19 did not demonstrate a corresponding structural abnormality in this region. No additional neuroradiological review or follow‐up imaging was performed; therefore, the nature of the CT finding remained indeterminate and it was not considered evidence of a definite structural cerebellar lesion (Figure 1B). Cranial and cervicocephalic CT angiography showed no severe stenosis of the major arteries.

**FIGURE 1:**
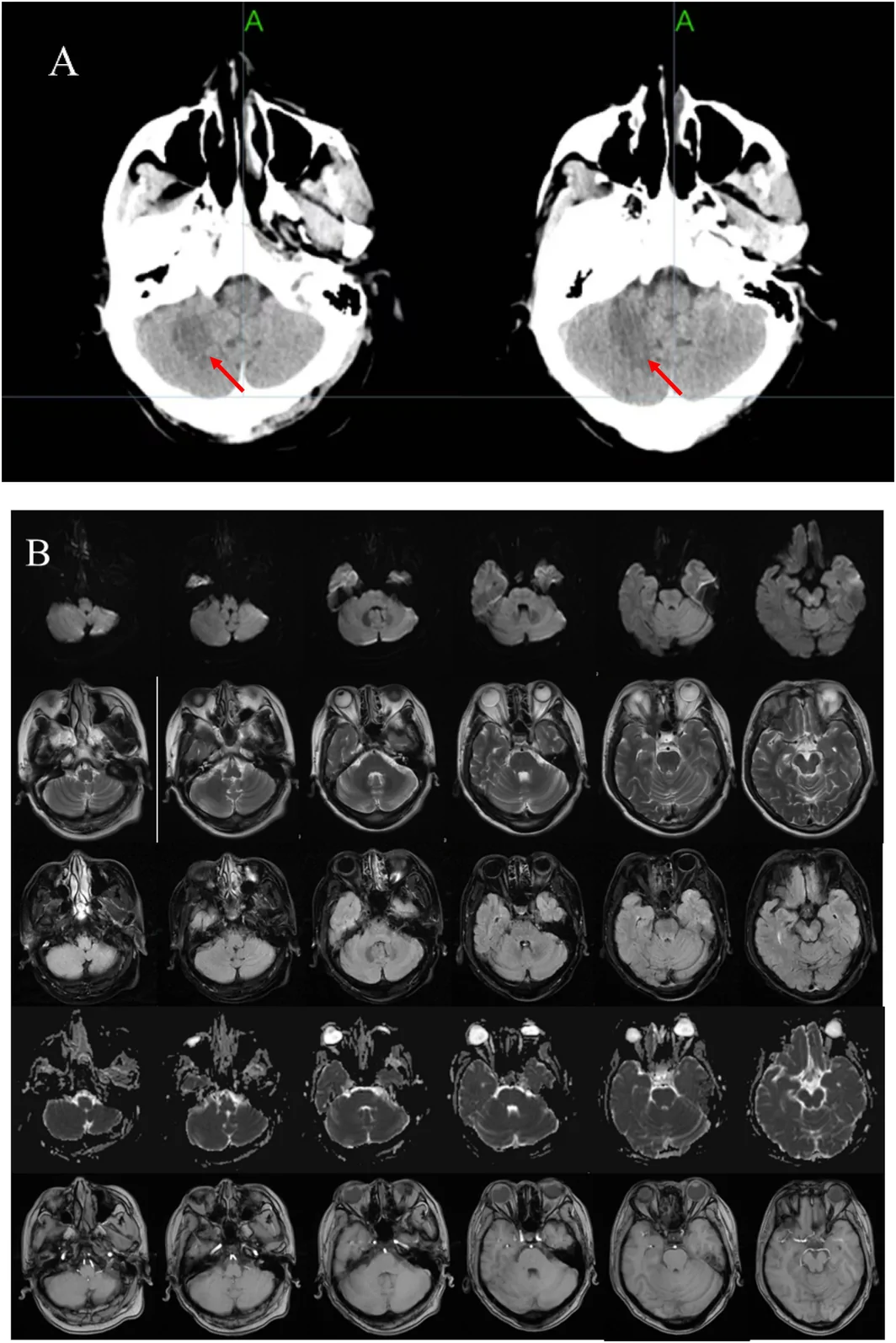
Neuroimaging. (A) Non‐contrast head CT showing a focal area of mildly decreased attenuation in the right cerebellar hemisphere (red arrow). (B) Subsequent brain MRI did not demonstrate a corresponding structural abnormality in the right cerebellar hemisphere.

Formal vestibular testing was performed on August 20, 2025, using the VertiPACS system (version 7.1.0; YouGeng Medical Equipment, Shanghai, China). For vHIT interpretation, VOR gains ≥ 0.8 for the horizontal semicircular canals and ≥ 0.7 for the anterior and posterior semicircular canals were considered within the laboratory reference range.

VNG showed left‐beating spontaneous nystagmus with a slow‐phase velocity (SPV) of 12.7°/s, which decreased to 3.9/s with visual fixation. The nystagmus increased on left gaze, consistent with Alexander's law. Saccadic testing was normal, smooth pursuit was Type I with superimposed left‐beating nystagmus, and horizontal optokinetic nystagmus gain was > 75% bilaterally. vHIT showed a markedly reduced VOR gain in the right horizontal semicircular canal (0.52) with overt corrective saccades. The VOR gains of the right anterior, left anterior, left horizontal, left posterior, and right posterior semicircular canals were 0.72, 0.93, 0.97, 0.97, and 0.86, respectively (Figure 2A).

**FIGURE 2:**
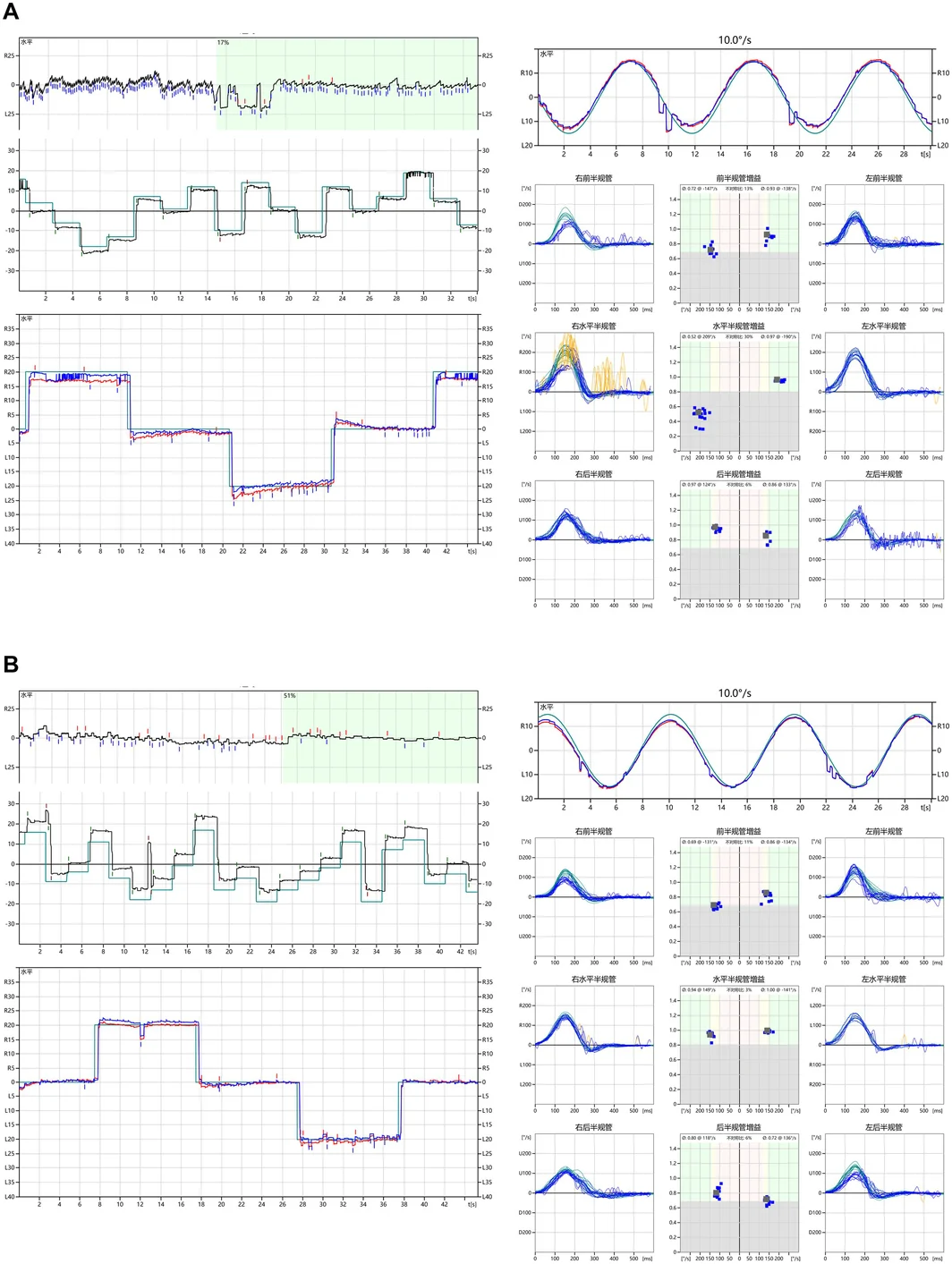
Vestibular testing. (A) On August 20, VNG showed left‐beating spontaneous nystagmus with a slow‐phase velocity (SPV) of 12.7/s, which decreased to 3.9/s with visual fixation. vHIT showed a markedly reduced VOR gain in the right horizontal semicircular canal (0.52) with overt corrective saccades. The VOR gains of the right anterior, left anterior, left horizontal, left posterior, and right posterior semicircular canals were 0.72, 0.93, 0.97, 0.97, and 0.86, respectively. Smooth pursuit was Type I with superimposed left‐beating nystagmus, and gaze testing showed enhancement of the left‐beating nystagmus on left gaze. (B) On September 8, VNG showed square‐wave jerks without clear spontaneous nystagmus. The VOR gains of the right anterior, left anterior, right horizontal, left horizontal, left posterior, and right posterior semicircular canals were 0.69, 0.86, 0.94, 1.00, 0.80, and 0.72, respectively, with no overt corrective saccades. Saccadic latency and accuracy were normal, smooth pursuit remained Type I, and gaze testing showed square‐wave jerks.

Taken together, the sustained vertigo, direction‐fixed and fixation‐suppressible spontaneous nystagmus, and unilateral horizontal‐canal VOR deficit constituted a peripheral‐appearing acute vestibular syndrome compatible with right acute unilateral vestibulopathy (AUVP)/vestibular neuritis (VN). A working diagnosis of right AUVP/VN was therefore made. Treatment included prednisone 10 mg three times daily for 5 days and adenosylcobalamin. Vestibular rehabilitation was recommended but declined by the patient. The continuous vertigo gradually resolved and had completely subsided by August 26, when she was discharged.

### 2.4 Recurrent Episodes and Second Admission

Approximately 5 days after discharge, the patient developed two recurrent episodes of vertigo, each lasting 2–3 h. These episodes interfered with her daily activities and required bed rest for symptom relief. They frequently occurred after meals and were not accompanied by nausea, vomiting, or gait unsteadiness. She requested readmission on September 5, 2025. Given the recurrent vestibular episodes and the emerging possibility of a migraine‐related vestibular disorder, flunarizine 5 mg nightly was initiated on September 8, 2025.

On September 12, she experienced another approximately 2‐h episode of vertigo after a meal. While looking out of the window, she perceived the clouds as drifting and experienced photophobia and phonophobia. The symptoms resolved after she rested in a darkened room. No nausea or vomiting occurred.

Repeat vestibular assessment was performed on September 8 using the same system and examination protocol and by the same examiner (Figure 2B). VNG showed square‐wave jerks without clear spontaneous nystagmus. Saccadic latency and accuracy remained normal, smooth pursuit remained Type I, and bilateral horizontal optokinetic gain was > 75%. Repeat vHIT showed substantial improvement in the previously reduced right horizontal‐canal VOR gain from 0.52 to 0.94, with no overt corrective saccades. The VOR gains of the right anterior, left anterior, left horizontal, left posterior, and right posterior semicircular canals were 0.69, 0.86, 1.00, 0.80, and 0.72, respectively. The right anterior‐canal VOR gain was marginally below the laboratory reference threshold of 0.70.

Gastroscopy demonstrated mild non‐atrophic gastritis. The Dizziness Handicap Inventory (DHI) score was 12 (Physical, 2; Emotional, 0; Functional, 10), the Self‐Rating Anxiety Scale score was 26, and the Self‐Rating Depression Scale score was 28.

The subsequent recurrent vestibular episodes, particularly the episode associated with photophobia and phonophobia, raised the possibility of a migraine‐related vestibular disorder and prompted reassessment of the initial diagnostic formulation. Based on the subsequent clinical course and the Bárány Society/International Headache Society diagnostic framework, the later recurrent vestibular syndrome was considered probable vestibular migraine (pVM). During the subsequent 2‐month follow‐up, three additional vestibular episodes were documented, all of which were accompanied by photophobia and phonophobia. Overall, four of the six recurrent vestibular episodes were associated with photophobia and phonophobia. The longitudinal clinical course, including the initial acute vestibular syndrome, serial vestibular assessments, recurrent episodes, and subsequent diagnostic reassessment, is illustrated in Figure 3. Details of the individual vestibular episodes are summarized in Table 1.

**FIGURE 3:**
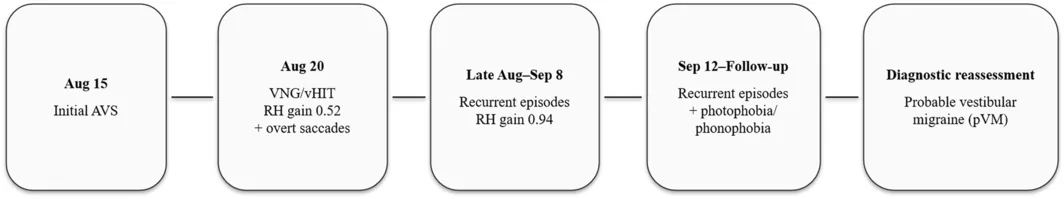
Clinical timeline of the patient's vestibular syndrome and diagnostic reassessment. The patient initially presented with an acute vestibular syndrome (AVS). Serial vestibular testing showed improvement in the previously reduced right horizontal‐canal vestibulo‐ocular reflex gain. During follow‐up, recurrent vestibular episodes emerged, with later episodes accompanied by photophobia and phonophobia. Based on the longitudinal clinical pattern, the recurrent vestibular syndrome was subsequently considered probable vestibular migraine (pVM).

**TABLE 1:** Summary of vestibular episodes and longitudinal clinical evolution.

Episode/period	Date/timing	Duration	Vestibular symptoms	Associated symptoms	Migraine‐associated features	Clinical context
Initial episode	15‐Aug‐25	Sustained for several hours; continuous symptoms subsequently persisted	Sudden‐onset vertigo described as marked swaying	Nausea and multiple episodes of vomiting; no cochlear symptoms	No headache, visual aura, photophobia, or phonophobia reported	Initial AVS; subsequent vestibular testing showed a peripheral‐appearing right unilateral vestibular deficit
Recurrent episode 1	~5 days after discharge	2–3 h	Recurrent vertigo	No nausea, vomiting, or gait unsteadiness	Not documented	Occurred before second admission; frequently associated temporally with meals
Recurrent episode 2	Before September 5, 2025	2–3 h	Recurrent vertigo	No nausea, vomiting, or gait unsteadiness	Not documented	Occurred before second admission; frequently associated temporally with meals
Recurrent episode 3	12‐Sep‐25	~2 h	Vertigo; perceived clouds as drifting while looking out of the window	No nausea or vomiting; symptoms improved after resting in a darkened room	Photophobia and phonophobia	Occurred 4 days after initiation of flunarizine
Recurrent episode 4	During subsequent 2‐month follow‐up	Several hours to half a day	Recurrent vertigo	Required rest for symptom relief	Photophobia and phonophobia	Follow‐up episode
Recurrent episode 5	During subsequent 2‐month follow‐up	Several hours to half a day	Recurrent vertigo	Required rest for symptom relief	Photophobia and phonophobia	Follow‐up episode
Recurrent episode 6	During subsequent 2‐month follow‐up	Several hours to half a day	Recurrent vertigo	Required rest for symptom relief	Photophobia and phonophobia	Follow‐up episode

## 3 Methods

This patient initially presented with an acute vestibular syndrome (AVS), characterized by acute‐onset sustained vertigo, nausea and vomiting, and spontaneous nystagmus. The initial vestibular findings were compatible with a right acute unilateral vestibulopathy (AUVP), commonly termed vestibular neuritis (VN) in the absence of acute audiological symptoms. Specifically, the patient exhibited direction‐fixed left‐beating spontaneous nystagmus that obeyed Alexander's law and was suppressed by visual fixation, together with a markedly reduced vestibulo‐ocular reflex (VOR) gain in the right horizontal semicircular canal (0.52) and overt corrective saccades. No acute hearing loss, tinnitus, aural fullness, or other cochlear symptoms were reported. These findings were consistent with the Bárány Society diagnostic framework for AUVP and supported right AUVP/VN as the initial working diagnosis [8, 11, 12]. Nevertheless, a peripheral‐appearing AVS does not completely exclude a central cause, particularly posterior circulation stroke involving the brainstem or cerebellum [2, 3, 13, 14, 15]. In this patient, the bedside head‐impulse test could not be reliably interpreted because of suboptimal cooperation, and a complete HINTS examination, including a clearly documented test of skew, was not available. Emergency CT showed no definite intracranial hemorrhage, infarction, or space‐occupying lesion, although a focal area of mildly decreased attenuation was noted in the right cerebellar hemisphere and follow‐up imaging was recommended. Subsequent brain MRI performed approximately 4 days after symptom onset demonstrated bilateral frontal and parietal subcortical and periventricular ischemic white‐matter changes (Fazekas score 2), without a reported corresponding structural abnormality in the right cerebellar hemisphere. Thus, the focal cerebellar CT finding was not corroborated by the subsequent MRI. These imaging findings reduced the probability of an acute posterior circulation structural lesion but were interpreted in conjunction with the clinical and vestibular findings rather than as categorically excluding a central etiology.

The subsequent course required further diagnostic reassessment. Symptoms and spontaneous nystagmus in AUVP/VN may improve over days to weeks through a combination of peripheral recovery and central vestibular compensation, whereas recovery of vHIT gain shows substantial interindividual variability and may depend on the severity of the initial vestibular deficit [10, 11, 16, 17, 18]. Therefore, the resolution of spontaneous nystagmus and substantial improvement in the previously reduced right horizontal‐canal VOR gain (from 0.52 to 0.94) approximately 24 days after symptom onset and 19 days after the initial vestibular assessment cannot, by themselves, be considered evidence against AUVP/VN or be used to distinguish AUVP/VN from probable vestibular migraine (pVM). Rather, the subsequent transition from a sustained acute vestibular syndrome to recurrent episodic vertigo, particularly with the later emergence of photophobia and phonophobia, prompted reconsideration of the overall diagnostic framework. The relationship between the initial AUVP/VN‐compatible episode and the later migraine‐associated recurrent episodes is addressed further in the Discussion.

## 4 Conclusion and Results (Outcome and Follow‐Up)

Flunarizine 5 mg nightly was initiated on September 8 as prophylactic treatment for the recurrent vestibular syndrome. Before initiation of flunarizine, two recurrent vestibular episodes had been documented after the first discharge. One additional episode occurred on September 12, 4 days after initiation of flunarizine, and three further episodes were documented during the subsequent 2‐month follow‐up. Because the pretreatment and posttreatment observation periods differed and treatment had been initiated only 4 days before the September 12 episode, treatment efficacy could not be reliably assessed from attack frequency. Accordingly, the clinical response to flunarizine was not considered diagnostic evidence for pVM. Lifestyle modification and vestibular rehabilitation were also recommended as part of individualized long‐term management [19, 20, 21, 22].

## 5 Discussion

Vestibular migraine (VM) is a clinically heterogeneous disorder that may present with spontaneous, positional, visually induced, or head‐motion–induced vestibular symptoms, with individual episodes typically lasting from 5 min to 72 h [6]. Although the diagnosis of definite VM and probable vestibular migraine (pVM) is primarily clinical, oculomotor, and vestibular abnormalities are not uncommon, particularly during symptomatic periods [23]. Spontaneous nystagmus during VM attacks may be horizontal, vertical, or torsional, with horizontal nystagmus being frequently observed. Previous studies have reported spontaneous nystagmus on videonystagmography (VNG) in approximately 21.7% of patients with VM, reduced vHIT gain in at least one semicircular canal in approximately 21.7%, and corrective saccades in approximately 18.3% [23]. More recent studies comparing symptomatic and symptom‐free phases have further demonstrated that vestibular findings may vary over time [24, 25]. These observations illustrate the heterogeneity of vestibular involvement in VM; however, they do not establish diagnostic specificity. In particular, reduced vHIT gain or corrective saccades neither establish nor exclude VM, and unilateral vestibular hypofunction should therefore be interpreted within the overall clinical context.

This distinction is particularly relevant when differentiating VM/pVM from acute unilateral vestibulopathy (AUVP)/vestibular neuritis (VN). The initial presentation in our patient—sustained acute vertigo, direction‐fixed left‐beating spontaneous nystagmus following Alexander's law and suppressed by visual fixation, together with a markedly reduced right horizontal‐canal VOR gain (0.52) and overt corrective saccades—was compatible with a right peripheral unilateral vestibular deficit and therefore supported AUVP/VN as the initial working diagnosis [8, 10, 11, 12]. However, the subsequent disappearance of spontaneous nystagmus and substantial improvement in the previously reduced right horizontal‐canal VOR gain (from 0.52 to 0.94) approximately 24 days after symptom onset and 19 days after the initial vestibular assessment should not be interpreted as being inconsistent with AUVP/VN. Spontaneous nystagmus after acute unilateral vestibular loss may diminish over days to weeks through a combination of peripheral recovery and central vestibular compensation, while residual nystagmus may persist longer when visual fixation is removed [10, 17]. Recovery of vHIT gain is also variable and is influenced by the magnitude of the initial vestibular deficit; abnormalities may persist for months in some patients, whereas substantial recovery may occur earlier in others [10, 11, 17, 18, 23]. Consequently, no established temporal threshold allows the observed improvement in vHIT gain over a 19‐day interval between assessments to reliably distinguish VM/pVM from AUVP/VN.

The serial vestibular findings in this patient should therefore be interpreted descriptively rather than as a diagnostic biomarker for pVM. On follow‐up, the previously observed spontaneous nystagmus was no longer evident, and the previously reduced right horizontal‐canal VOR gain had increased from 0.52 to 0.94, with no overt corrective saccades; square‐wave jerks were observed on VNG. Accordingly, the follow‐up VNG represented a changed oculomotor pattern rather than complete “VNG normalization.” Square‐wave jerks can be nonspecific, particularly in older adults, but may also occur in association with central ocular‐motor dysfunction. Their presence does not establish a central mechanism in this patient, but it further argues against characterizing the entire VNG examination as normal. Similarly, although central vestibular hyperexcitability, altered sensory integration, and cortical or brainstem network dysfunction have been proposed as mechanisms underlying vestibular abnormalities in VM [26, 27], the present case cannot distinguish transient central modulation from peripheral vestibular recovery, central compensation, or measurement variability. Therefore, the observed changes should not be attributed to a specific pathophysiological mechanism.

The Bárány Society and the International Headache Society have established diagnostic criteria for definite VM and pVM [6]. Definite VM requires at least five episodes of moderate or severe vestibular symptoms lasting 5 min to 72 h, a current or previous history of migraine with or without aura, and migraine features during at least 50% of vestibular episodes, with symptoms not better accounted for by another diagnosis. For pVM, at least five qualifying vestibular episodes are required, but only one of the two migraine‐related criteria—a history of migraine or migraine features during the vestibular episodes—is required [6]. Following resolution of the initial sustained acute vestibular syndrome, the patient experienced six subsequent recurrent vestibular episodes: two before the second admission, one during the second hospitalization, and three during the subsequent 2‐month follow‐up. These episodes lasted approximately 2 h to half a day. The two episodes before the second admission interfered with daily activities and required bed rest, supporting at least moderate severity. Four of the six recurrent vestibular episodes were accompanied by both photophobia and phonophobia, thereby meeting the requirement for migraine features during at least 50% of the vestibular episodes. However, the patient had no documented current or previous history of migraine with or without aura and therefore did not fulfill the criteria for definite VM. Taken together, the later recurrent vestibular syndrome was considered consistent with the Bárány Society/International Headache Society diagnostic criteria for pVM.

Importantly, classifying the later recurrent vestibular syndrome as pVM does not retrospectively establish that the initial acute peripheral‐appearing vestibular syndrome was a manifestation of the same disorder. Several explanations remain plausible. The patient may have experienced an initial episode of AUVP/VN followed by pVM; alternatively, pVM may have been associated with transient peripheral‐appearing vestibular abnormalities. Coexisting vestibular disorders or variability related to serial vestibular measurements also cannot be excluded. The available findings from a single case do not allow these possibilities to be reliably distinguished. Thus, the later emergence of migraine‐associated features should be understood as changing the diagnostic probability and prompting reassessment rather than replacing the initial AUVP/VN‐compatible diagnosis or proving a common etiology for all episodes.

The principal clinical value of this case therefore lies in the longitudinal evolution of the vestibular phenotype rather than in the diagnostic specificity of any individual vestibular test. Serial VNG and vHIT can objectively document changes in vestibular and oculomotor findings, but their results should be integrated with the temporal pattern of symptoms, associated migraine features, neurological examination, and neuroimaging. In patients whose presentation evolves from a sustained peripheral‐appearing AVS to recurrent episodic vertigo with migraine‐associated features, reassessment of the differential diagnosis is warranted. Dynamic vestibular testing may contribute to this reassessment by documenting changing vestibular physiology, but it should not be used in isolation to distinguish pVM from AUVP/VN.

## Author Contributions

**Ping Zhong:** project administration. **Xiaocui Kang:** funding acquisition, writing – original draft. **Shiyu Jiang:** data curation. **Ying Wu:** data curation. **Siyang Dai:** data curation. **Zhenyu Wei:** data curation.

## Funding

This study was supported by grants from the 2023 Joint Project of the Yangpu District Science and Technology Commission and Health Commission (YPZYQ202302), and the Institutional Project of Shidong Hospital, Yangpu District, Shanghai (YJYB03).

## Consent

Written informed consent was obtained from the patient for publication of this case report and accompanying images.

## Conflicts of Interest

The authors declare no conflicts of interest.

## Data Availability

The data that support the findings of this study are available on request from the corresponding author. The data are not publicly available due to privacy or ethical restrictions.

## References

[1] K. D. Choi , H. Lee , and J. S. Kim , “Vertigo in Brainstem and Cerebellar Strokes,” Current Opinion in Neurology 26, no. 1 (2013): 90–95, 10.1097/WCO.0b013e32835c5edd. 23254553

[2] H. Lee , “Isolated Vascular Vertigo,” Journal of Stroke 16, no. 3 (2014): 124–130, 10.5853/jos.2014.16.3.124. PMC4200599 25328871

[3] J. C. Kattah , A. V. Talkad , D. Z. Wang , Y. H. Hsieh , and D. E. Newman‐Toker , “HINTS to Diagnose Stroke in the Acute Vestibular Syndrome,” Stroke 40, no. 11 (2009): 3504–3510, 10.1161/STROKEAHA.109.551234. PMC4593511 19762709

[4] A. A. Tarnutzer , A. L. Berkowitz , K. A. Robinson , et al., “Does My Dizzy Patient Have a Stroke?,” CMAJ: Canadian Medical Association Journal 183, no. 9 (2011): E571–E592. 10.1503/cmaj.100174 PMC3114934 21576300

[5] M. von Brevern and T. Lempert , “Vestibular Migraine,” Handbook of Clinical Neurology 137 (2016): 301–316, 10.1016/B978-0-444-63437-5.00022-4. 27638080

[6] T. Lempert , J. Olesen , J. Furman , et al., “Vestibular Migraine: Diagnostic Criteria (Update): Literature Update 2021,” Journal of Vestibular Research: Equilibrium & Orientation 32, no. 1 (2022): 1–6. 10.3233/VES-201644 PMC9249276 34719447

[7] A. Radtke , T. Lempert , M. A. Gresty , et al., “Migraine and Ménière's Disease: Is There a Link?,” Neurology 59, no. 11 (2002): 1700–1704. 10.1212/01.wnl.0000036903.22461.39 12473755

[8] M. Strupp , A. Bisdorff , J. Furman , et al., “Acute Unilateral Vestibulopathy/Vestibular Neuritis: Diagnostic Criteria. Consensus Document of the Committee for the Classification of Vestibular Disorders of the Bárány Society,” Journal of Vestibular Research: Equilibrium & Orientation 32, no. 5 (2022): 389–406. 10.3233/VES-220201 PMC9661346 35723133

[9] J. S. Kim , “When the Room Is Spinning: Experience of Vestibular Neuritis by a Neurotologist,” Frontiers in Neurology 11 (2020): 157, 10.3389/fneur.2020.00157. PMC7062794 32194499

[10] L. A. McGarvie , H. G. MacDougall , I. S. Curthoys , and G. M. Halmagyi , “Spontaneous Recovery of the Vestibulo‐Ocular Reflex After Vestibular Neuritis,” Frontiers in Neurology 11 (2020): 732. 10.3389/fneur.2020.00732 PMC7399092 32849204

[11] G. M. Halmagyi , L. Chen , H. G. MacDougall , K. P. Weber , L. A. McGarvie , and I. S. Curthoys , “The Video Head Impulse Test,” Frontiers in Neurology 8 (2017): 258, 10.3389/fneur.2017.00258. PMC5465266 28649224

[12] M. Strupp and T. Brandt , “Vestibular Neuritis,” Seminars in Neurology 29, no. 5 (2009): 509–519, 10.1055/s-0029-1241040. 19834862

[13] J. S. Kim , D. E. Newman‐Toker , K. A. Kerber , et al., “Vascular Vertigo and Dizziness: Diagnostic Criteria. Consensus Document of the Committee for the Classification of Vestibular Disorders of the Bárány Society,” Journal of Vestibular Research 32, no. 3 (2022): 205–222. 10.3233/VES-210169 PMC9249306 35367974

[14] H. A. Kim and H. Lee , “Recent Advances in Central Acute Vestibular Syndrome of a Vascular Cause,” Journal of the Neurological Sciences 321, no. 1–2 (2012): 17–22, 10.1016/j.jns.2012.07.055. 22906582

[15] D. E. Newman‐Toker , J. C. Kattah , J. E. Alvernia , and D. Z. Wang , “Normal Head Impulse Test Differentiates Acute Cerebellar Strokes From Vestibular Neuritis,” Neurology 70, no. 24 Pt 2 (2008): 2378–2385, 10.1212/01.wnl.0000314685.01433.0d. 18541870

[16] J. Sjögren , P. A. Fransson , M. L. Å. Karlberg , M. Magnusson , and F. Tjernström , “Vestibular Recovery and Central Compensation After Acute Unilateral Vestibulopathy—Changes in Saccadic Response Patterns and Gains Over Time,” Frontiers in Neurology 17 (2026): 1832160, 10.3389/fneur.2026.1832160. PMC13152781 42111065

[17] C. C. Chen , A. K. Bery , and T. P. Chang , “Weak Nystagmus in the Dark Persists for Months After Acute Unilateral Vestibular Loss,” Frontiers in Neurology 14 (2023): 1327735, 10.3389/fneur.2023.1327735. PMC10754966 38162452

[18] H. G. MacDougall and I. S. Curthoys , “Plasticity During Vestibular Compensation: The Role of Saccades,” Frontiers in Neurology 3 (2012): 21, 10.3389/fneur.2012.00021. PMC3289127 22403569

[19] S. C. Beh , “Vestibular Migraine: How to Sort It Out and What to Do About It,” Journal of Neuro‐Ophthalmology 39, no. 2 (2019): 208–219, 10.1097/WNO.0000000000000791. 31094996

[20] D. Smyth , Z. Britton , L. Murdin , Q. Arshad , and D. Kaski , “Vestibular Migraine Treatment: A Comprehensive Practical Review,” Brain 145, no. 11 (2022): 3741–3754, 10.1093/brain/awac264. PMC9679161 35859353

[21] H. Chu , Y. Wang , X. Ling , et al., “Prophylactic Treatments for Vestibular Migraine: A Systematic Review and Network Meta‐Analysis,” Frontiers in Pharmacology 14 (2023): 1332973. 10.3389/fphar.2023.1332973 PMC10771287 38186654

[22] K. Chrysouli and S. Karamagkiolas , “Recent Trends in Treatment of Vestibular Migraine: A Systematic Review,” Indian Journal of Otolaryngology and Head & Neck Surgery 76, no. 3 (2024): 2237–2243, 10.1007/s12070-024-04546-6. PMC11169333 38883465

[23] S. Waissbluth , V. Sepúlveda , J. S. Leung , and J. Oyarzún , “Vestibular and Oculomotor Findings in Vestibular Migraine Patients,” Audiology Research 13, no. 4 (2023): 615–626, 10.3390/audiolres13040053. PMC10452030 37622929

[24] Y. Li , Y. Wang , M. Chen , R. Jiang , and Y. Ju , “Eye Movement Abnormalities During Different Periods in Patients With Vestibular Migraine,” Journal of Pain Research 16 (2023): 3583–3590, 10.2147/JPR.S422255. PMC10614654 37908779

[25] F. Batinović , D. Sunara , N. Pleić , et al., “Clinical Features, Video Head Impulse Test, and Subjective Visual Vertical of Acute and Symptom‐Free Phases in Patients With Definite Vestibular Migraine,” Biomedicine 13, no. 4 (2025): 825, 10.3390/biomedicines13040825. PMC12024932 40299426

[26] J. H. Shin , Y. K. Kim , H. J. Kim , and J. S. Kim , “Altered Brain Metabolism in Vestibular Migraine,” Cephalalgia 34, no. 1 (2014): 58–67. 10.1177/0333102413498940 23918837

[27] A. Russo , V. Marcelli , F. Esposito , et al., “Abnormal Thalamic Function in Patients With Vestibular Migraine,” Neurology 82, no. 23 (2014): 2120–2126, 10.1212/WNL.0000000000000496. 24814847

